# The clinical and prognostic values of optic nerve sheath diameter and optic nerve sheath diameter/eyeball transverse diameter ratio in comatose patients with supratentorial lesions

**DOI:** 10.1186/s12883-021-02285-7

**Published:** 2021-07-02

**Authors:** Sha Zhu, Chao Cheng, Dianjiang Zhao, Yuanli Zhao, Xianzeng Liu, Jun Zhang

**Affiliations:** 1 Department of Neurology, Peking University People’s Hospital, No. 11 South Avenue, Xi Zhi Men Xicheng District, Beijing, 100044 China; 2 Department of Radiology, Peking University International Hospital, Beijing, China; 3 Department of Neurosurgery, Peking University International Hospital, Beijing, China; 4 Department of Neurosurgery, Beijing TianTan Hospital, Beijing, China; 5 Department of Neurology, Peking University International Hospital, No. 1 Life Science Park Life Road, Changping District, Beijing, 102206 China

**Keywords:** Optic nerve sheath diameter, Eyeball transverse diameter, Coma, Intracranial pressure, Prognosis

## Abstract

**Background:**

The optic nerve sheath diameter (ONSD) and ONSD/eyeball transverse diameter (ETD) ratio have been proven to be correlated with intracranial pressure. This study aimed to evaluate the prognostic roles of ONSD and the ONSD/ETD ratio in comatose patients with supratentorial lesions and to determine the relationship of these two indices with the prognosis of such patients.

**Methods:**

A total of 54 comatose patients with supratentorial lesions and 50 healthy controls were retrospectively included in this study. ONSD and ETD were measured by unenhanced computed tomography (CT). The differences in ONSD and the ONSD/ETD ratio between the two groups were compared. The prognosis of comatose patients was scored using the Glasgow Outcome Scale (GOS) at the 3-month follow-up, and these patients were classified into good (GOS score ≥ 3) and poor (GOS score < 3) prognosis groups. The differences in ONSD and the ONSD/ETD ratio were compared between comatose patients with good prognoses and those with poor prognoses.

**Results:**

The ONSD and ONSD/ETD ratios in the comatose patients were 6.30 ± 0.60 mm and 0.27 ± 0.03, respectively, and both were significantly greater than those in the healthy controls (5.10 ± 0.47 mm, t = 11.426, *P* < 0.0001; 0.22 ± 0.02, t = 11.468, *P* < 0.0001; respectively). ONSD in patients with poor prognosis was significantly greater than that in patients with good prognosis (6.40 ± 0.56 vs. 6.03 ± 0.61 mm, t = 2.197, *P* = 0.032). The ONSD/ETD ratio in patients with poor prognosis was significantly greater than that in patients with good prognosis (0.28 ± 0.02 vs. 0.26 ± 0.03, t = 2.622, *P* = 0.011). The area under the receiver operating characteristic (ROC) curve, used to predict the prognosis of comatose patients, was 0.650 (95% confidence interval (*CI*): 0.486–0.815, *P* = 0.078) for ONSD and 0.711 (95% *CI*: 0.548–0.874, *P* = 0.014) for the ONSD/ETD ratio.

**Conclusions:**

The ONSD and ONSD/ETD ratios were elevated in comatose patients. The ONSD/ETD ratio might be more valuable than ONSD in predicting the prognoses of comatose patients with supratentorial lesions.

## Background

Coma is characterized by a severe disruption in arousal and awareness and is associated with a high mortality rate. Diverse causes of coma have been reported, such as hypoxic ischaemic encephalopathy (HIE), traumatic brain injury (TBI), cerebrovascular disease, brain tumours, infection, inflammation, etc. [1]. Currently, a large number of methods are used to predict the prognosis of comatose patients, including electroencephalogram, somatosensory evoked potential, transcranial Doppler ultrasound, serology, etc.

Supratentorial lesions refer to diseases above the tentorium of the cerebellum that might elevate intracranial pressure (ICP). From pathological examinations, it was reported that supratentorial lesions can cause brain shift, followed by herniation, brainstem compression, and death [1]. Brain herniation occurs when increased ICP causes abnormal protrusion of brain tissue through openings in rigid intracranial barriers (e.g., the tentorial notch). ICP could be directly related to the prognosis of comatose patients. Therefore, monitoring of ICP is important for comatose patients with supratentorial lesions, assisting clinicians in making robust decisions.

External ventricular drains (EVDs) are frequently utilized to accurately monitor and treat ICP, but they are invasive and costly and can lead to a series of complications, such as intracranial infection and induced cerebral haemorrhage, thereby restricting their clinical application.

Noninvasive methods for measuring and evaluating ICP have been developed but have not been demonstrated to be sufficiently reliable to use on a routine basis. Since the optic nerve sheath communicates with the intracranial subarachnoid space [2], and the ICP level can be indirectly determined by the optic nerve sheath diameter, measurement of ONSD has emerged as a noninvasive technique in recent years. Currently, ONSD can be evaluated with ultrasonography, magnetic resonance imaging (MRI), and CT scans. Among these methods, CT is more appropriate for diagnosing comatose patients because it can promptly detect the cause of coma and evaluate the development of the disease. Previous studies have shown that ONSD is strongly correlated with ICP level; when ICP level increases, the elevation of ONSD is noteworthy, and vice versa [3–6]. The prognostic value of ONSD has been reported in several studies [3, 5, 7, 8]. A number of scholars have demonstrated that ONSD is strongly correlated with ETD in healthy individuals [9]. The ONSD/ETD ratio has shown better predictive value than ONSD in ICP monitoring [8], although few data are available regarding its prognostic value.

The present study aimed to assess the prognostic roles of ONSD and the ONSD/ETD ratio in comatose patients with supratentorial lesions and to determine the relationship between these two indices and the prognosis of comatose patients.

## Methods

### Study design and clinical collection

This retrospective study was approved by the Ethics Committee of Peking University International Hospital (Approval No. 2021–001 (BMR)). Comatose patients with supratentorial lesions who were admitted to the Neurological Intensive Care Unit (NICU) of Peking University International Hospital (Beijing, China) from August 2015 to November 2020 were enrolled in the study. The inclusion criteria were as follows: 1) patients aged ≥ 18 and ≤ 80 years old; 2) supratentorial lesions detected by unenhanced CT, including acute cerebral infarction (ACI), cerebral haemorrhage (CH), subarachnoid haemorrhage (SAH), and TBI; and 3) comatose patients with Glasgow coma scale (GCS) scores ≤ 8 upon admission. All of the comatose patients had indirect CT signs of intracranial hypertension, such as midline shift, cerebral oedema, compressed cisterns, small ventricles, and sulcal effacement. The exclusion criteria were as follows: 1) history of glaucoma, thyroid-associated ophthalmopathy, or optic nerve diseases; 2) combined ocular and optic nerve injuries at admission; 3) lumbar puncture performed within 2 weeks before the measurement of ONSD and a history of SAH; and 4) serious complications that could affect life expectancy (e.g., haematopathy, tumour, etc.).

The control group included 50 subjects who were admitted to our outpatient department for physical examinations and were matched with comatose patients in terms of age and sex parameters. In this group, acute intracranial lesions were excluded by brain CT scans, and the physical examinations were unremarkable regarding signs and symptoms of intracranial hypertension. Patients with ophthalmic diseases and a history of SAH were excluded.

The study subjects’ clinical data were reviewed and collected, including age, sex, weight, body mass index (BMI), and mean arterial pressure (MAP). Data related to primary disease, clinical history, GCS score, and operations during hospitalization that could affect ICP levels, such as haematoma clearance and decompressive craniectomy, were also recorded for the comatose patients.

### Measurement of ONSD and ETD

**Fig. 1:**
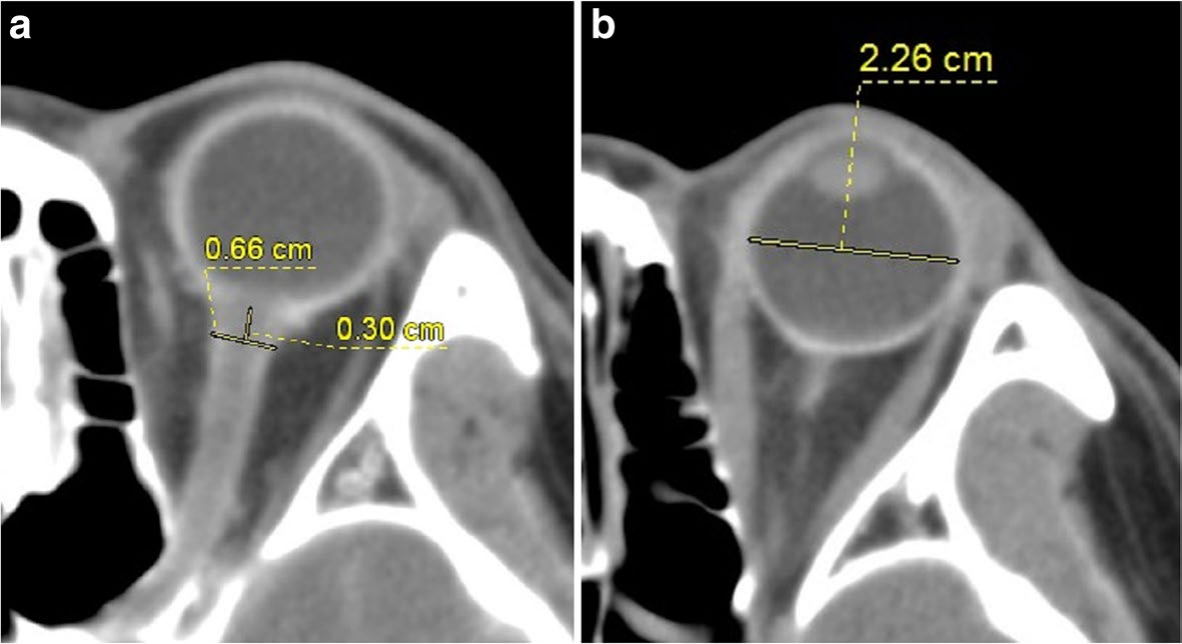
**A** Optic nerve sheath diameter (ONSD) measurement by head CT scan. **B** Eyeball transverse diameter (ETD) measurement by head CT scan

All of the comatose patients underwent head CT scans on the first day of coma onset for aetiology assessment. The CT scans were performed on a spiral scanner (64 row; Siemens Healthcare Diagnostics Inc., Berlin, Germany) with a tube voltage of 120 kV, tube current of 200–300 mA, slice thickness of 2 mm, slice interval of 3 mm, and pitch of 1. Two experienced radiologists who were blinded to the clinical data interpreted the CT images independently. The ONSD and ETD were measured at a fixed mediastinal window (width, 300; level, 35) for the same contrast and brightness. The direction of the optic nerve was identified by three-dimensional reconstruction of brain CT data, and the ONSD was measured 3 mm behind the eyeball [3] (Fig. 1). ETD was measured from one side of the retina behind the lens to the other for the maximum diameter (Fig. 1). The values measured by two radiologists were averaged. All of the measurements of ONSD and ETD were performed bilaterally, and the mean value was considered to calculate the ONSD/ETD ratio.

### Outcome assessment

The comatose patients were assessed with the GOS score at the 3-month follow-up by telephone or face-to-face interviews. According to the GOS scores, the patients were divided into two groups: good prognosis (GOS 3, severe disability; GOS 4, moderate disability; GOS 5, return to normal life) and poor prognosis (GOS 1, death; GOS 2, vegetative state).

### Statistical analysis

SPSS software (IBM, Armonk, NY, USA), version 23.0, was used to perform statistical analyses. Continuous variables are expressed as the mean ± standard deviation (SD). Abnormally distributed data are presented as the median (interquartile range). Categorical variables are expressed as counts (percentages). The chi-square test was used to compare data, including sex, GCS score, aetiology, and clinical data. The independent-samples t-test was utilized to compare data, such as the age, weight, BMI, ONSD, and ONSD/ETD ratio. Nonparametric tests were used to compare height and MAP. The area under the ROC curve was employed to assess the prognostic value of the ONSD and ONSD/ETD ratio. A *P* value < 0.05 was considered statistically significant.

## Results

### Clinical characteristics

A total of 54 comatose patients with supratentorial lesions and 50 healthy controls were included in this study. The median GCS score at admission in the coma group was 3.5 (interquartile range: 3–6). The main causes of coma included ACI (*n* = 12), CH (*n* = 19), SAH (*n* = 13), and TBI (*n* = 10). The demographic characteristics of the comatose patients and healthy controls at baseline are summarized in Table 1.

**Table 1:** The demographic characteristics of the comatose patients and healthy controls at baseline

Characteristic	Coma group (*n* = 54)	Control group (*n* = 50)	χ^2^/t/z value	*P* value
Age [yr/d, mean (SD)	58.8 (17.4)	54.9 (17.0)	1.155	0.251
Sex, male [ n (%)]	27 (50.0)	24 (48.0)	0.042	0.847
Height [cm, mean (SD)]	165.7 (7.5)	164.0 (7.5)	-1.442	0.150
Weight [kg, mean (SD)]	66.2 (13.9)	67.3 (12.1)	-0.424	0.672
BMI [mean (SD)]	23.5(4.8)	24.9(3.4)	-1.753	0.083
MAP [mean (SD)]	86.2 (20.6)	90.6 (15.5)	-2.392	0.016

*SD* standard deviation, *BMI* body mass index, *MAP* mean arterial pressure

### Comparing the ONSD and ONSD/ETD ratio between the coma and control groups

The ONSDs in the coma group and control group were 6.30 ± 0.60 mm and 5.10 ± 0.47 mm, respectively (t = 11.426, *P* < 0.0001). In addition, the ONSD/ETD ratios in the coma group and control group were 0.27 ± 0.03 and 0.22 ± 0.02, respectively (t = 11.468, *P* < 0.0001).

### Outcomes of comatose patients and the associated factors

At the 3-month follow-up, 17 (31.5%) patients had good outcomes, while 37 (68.5%) patients had poor outcomes. Table 2 presents the results of the comparison between patients with good prognosis and those with poor prognosis. There were significant differences in the GCS score (χ2 = 28.834, *P* < 0.0001), ONSD (t = 2.197, *P* = 0.032), and ONSD/ETD ratio (t = 2.622, *P* = 0.011) between the two groups. There were no significant differences in age, sex, height, weight, BMI, MAP, aetiology, past history or surgical operation.

**Table 2:** Comparison of patients with good prognoses and those with poor prognoses

Characteristic	Good prognosis (*n* = 17)	Poor prognosis (*n* = 37)	χ^2^/t/z value	*P* value
Age, yr [ mean (SD)]	52.8 (21.4)	61.6 (14.8)	1.544	0.136
Sex, male [n (%)]	9(52.9)	18(48.6)	0.086	1.000
Height [cm, mean (SD)]	167.4 (7.3)	164.9 (7.6)	-1.398	0.165
Weight [kg, mean (SD)]	68.5 (13.2)	65.1 (14.3)	-0.830	0.410
BMI [mean (SD)]	23.4(6.70)	23.5(3.8)	0.061	0.952
MAP [mean (SD)]	93.8 (22.1)	82.7 (19.2)	-1.491	0.138
GCS score [n(%)]			28.834	< 0.0001
3	0	27		
4	2	2		
5	4	4		
6	4	0		
7	2	2		
8	5	2		
Aetiology [n(%)]			6.270	0.093
ACI	3	9		
CH	9	10		
SAH	1	12		
TBI	4	6		
Clinical history				
Hypertension	7	14	0.055	1.000
Diabetes	3	7	0.012	1.000
CHD	3	4	0.482	0.665
Stroke	4	4	1.493	0.243
ONSD [mm, mean (SD)]	6.03(0.61)	6.40(0.56)	2.197	0.032
ONSD/ETD, [mean (SD)]	0.26(0.03)	0.28(0.02)	2.622	0.011
Operation [n(%)]	8 (47.1)	10 (27.0)	2.103	0.215

*SD* standard deviation, *BMI* body mass index, *MAP* mean arterial pressure, *GCS* Glasgow coma scale, *CHD* coronary heart disease, *ACI* acute cerebral infarction, *CH* cerebral haemorrhage, *SAH* subarachnoid haemorrhage, *TBI* traumatic brain injury, *ONSD* optic nerve sheath diameter, *ETD* eyeball transverse diameter

### Predictive efficiency of ONSD and ONSD/ETD ratio for outcomes of comatose patients

**Fig. 2:**
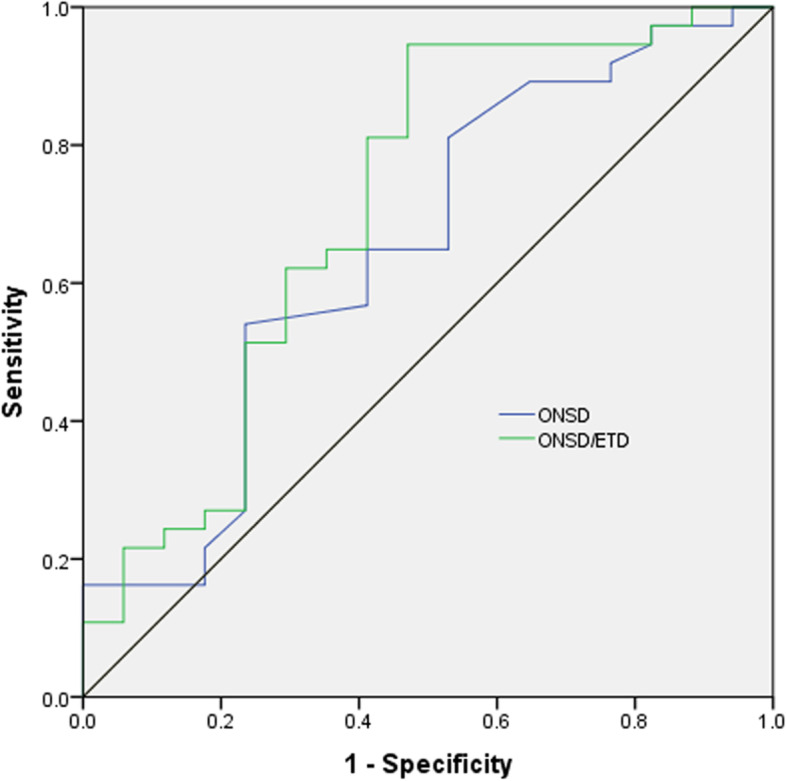
ROC curves for the efficiency of ONSD and ONSD/ETD ratio in predicting poor prognoses of comatose patients

The efficiency of ONSD and ONSD/ETD ratio in predicting poor prognoses of comatose patients is shown in Fig. 2. The area under the curve (AUC) for ONSD in predicting poor prognoses of comatose patients was 0.650 (95% confidence interval (*CI*): 0.486–0.815, *P* = 0.078), with sensitivity of 54.1% and specificity of 76.5% at a cut-off value of 6.4 mm; the value of AUC for ONSD/ETD ratio predicting poor prognoses of comatose patients was 0.711 (95% *CI*: 0.548–0.874, *P* = 0.014) at a cut-off value of 0.25, with sensitivity of 94.6% and specificity of 52.9%.

## Discussion

The results of the current study showed that the ONSD in the coma group was 6.30 ± 0.60 mm compared to 5.10 ± 0.47 mm in the control group. In addition, the ONSD/ETD ratio in the coma group was 0.27 ± 0.03 compared to 0.22 ± 0.02 in the control group. The ONSD and ONSD/ETD ratios were significantly greater in comatose patients with supratentorial lesions than in the healthy controls, which indirectly confirmed the relationship between ONSD and ICP. There were significantly greater ONSD and ONSD/ETD ratios in the poor prognosis group than in the good prognosis group. However, the performance of the ONSD/ETD ratio in predicting the outcomes of comatose patients was better than that of ONSD.

Monitoring of ICP is clinically significant for the prognosis and therapy of comatose patients. As a noninvasive method for monitoring ICP, ONSD has been used for 20 years and is expected to be an effective technique to predict the prognoses of patients. Conversely, the ONSD/ETD ratio was first described in 2014 and seemed to be more accurate in predicting ICP than ONSD alone [8, 9]. A number of scholars have demonstrated that ONSD increases with the elevation of ICP, and increased ICP is strongly correlated with the poor prognosis of patients with neurological disorders [7, 10–12]. It was found that ONSD was increased in 95% of patients with ICH or SAH, as indicated by CT, and ONSD was 6.6 ± 0.8 mm with a cut-off value of 5.5 mm, suggesting an elevated ICP [5]. Thus, the ONSD/ETD ratio might be more sensitive and specific for ICP assessment [6, 8].

A number of scholars have investigated the prognostic value of ONSD in patients with TBI and HIE [3, 7, 13, 14]. Shahan et al*.* pointed out that ONSD measured on the initial head CT scan was positively associated with the severity of blunt TBI [7]. Another study found that ONSD could be a good predictor of mortality (AUC: 0.805), and a cut-off ≥ 7.3 mm had sensitivity of 86.4% and specificity of 74.6% [13]. In addition, ONSD was strongly correlated with neurologic outcomes in patients with HIE [14]. Other scholars have demonstrated that ONSD showed potential prognostic value for poor neurological outcomes in patients undergoing hemicraniectomy [15]. Since the ONSD/ETD ratio is more clinically valuable than ONSD alone in predicting ICP [8], it might also be valuable in predicting the prognoses of comatose patients. However, to date, only a limited number of studies have concentrated on its prognostic value. It was previously found that changes in the ONSD/ETD ratio compared to baseline were predictive of late malignant progression in patients with malignant middle cerebral artery (MCA) infarction [16]. In cases of TBI without haemorrhage and those with haemorrhage, the ONSD/ETD ratios were 0.28 ± 0.05 and 0.29 ± 0.05, respectively, compared with 0.19 ± 0.02 in healthy adults, and an inverse correlation was identified between the ONSD/ETD ratio and the GOS score [17, 18]. An inverse correlation between the two indices has also been confirmed in nontraumatic intracranial haemorrhage [5]. The ONSD/ETD ratio showed excellent discrimination ability for identifying paediatric patients with headache and papilledema (AUC = 0.96) [19]. An ONSD greater than 5.25 mm and an ONSD/ETD ratio greater than 0.232 on initial CT could identify MCA stroke patients at high risk of developing malignant MCA syndrome [20]. However, ONSD seemed to be more valuable than the ONSD/ETD ratio in predicting the risk of developing large MCA infarcts [21]. The present study, for the first time, compared the efficiency of ONSD and the ONSD/ETD ratio in comatose patients with supratentorial lesions. The findings revealed that the ONSD/ETD ratio is more valuable in predicting the prognosis of comatose patients than ONSD. This result could be attributed to a smaller SD of the ONSD/ETD ratio compared to the ONSD measurement alone, contributing to the establishment of a significantly accurate method for detecting ICP.

In the present research, it was found that the “mediastinal” window was the best window for identifying the optic nerve, while a large number of previous studies used the “spine” window [5, 6, 9], the “abdomen” window [20], or an unspecified window when measuring ONSD. In addition, diverse distances and anatomical marks in the measurement could lead to great variability in the results of studies of ONSD. In this case, due to individual differences, monitoring of ONSD might be of great significance in determining abnormalities of ICP and predicting prognosis. Therefore, it is essential to establish a unified method to measure ONSD and determine the range of normal ONSD values.

To date, no study has determined whether ONSD is correlated with age, height, weight, BMI, MAP, etc. Some studies have shown that the ONSD/ETD ratio has no correlation with the aforementioned factors [22, 23]. Another study revealed that the ONSD/ETD ratio was not correlated with sex, height, weight, BMI, or head circumference [24]. Therefore, the ONSD/ETD ratio is more stable than ONSD alone. Similar to other nerves in the human body, optic nerve fibres deteriorate with ageing. However, the average diameter of axons and dural thickness could be increased with ageing [25, 26]. Therefore, a number of scholars have demonstrated that ONSD is approximately constant during the lifetime [9]. The current study showed that the aforementioned factors were not significantly different between the coma and control groups, except for MAP, which could be correlated with the haemodynamic instability of the comatose patients.

There are a number of limitations of the present research. First, the retrospective nature of the study and the small sample size might have led to inevitable bias. Second, asymmetric ONSD between the eyes has been described in both normal subjects and patients with intracranial hypertension [27, 28]. However, there have been no relevant studies of whether ETD is asymmetric, which could have led to uncertainty in the results. Third, the comatose patients in our study had different aetiologies, which might also have caused bias. Multicentre studies with larger sample sizes are needed to further evaluate ONSD in comatose patients with a specific aetiology and to determine the relationship of ONSD and the ONSD/ETD ratio with the prognoses of comatose patients.

## Conclusion

The present research showed that ONSD and ONSD/ETD ratios were significantly elevated in comatose patients with supratentorial lesions. In addition, the ONSD/ETD ratio might be more valuable than ONSD in predicting the prognosis of comatose patients. However, further studies with larger samples are needed to confirm our findings.

## Data Availability

The datasets used and/or analysed during the current study are available from the corresponding author on reasonable request.

## Abbreviations

ONSD: Optic nerve sheath diameter
ETD: Eyeball transverse diameter
GOS: Glasgow Outcome Scale
CT: Computed tomography
HIE: Hypoxic ischaemic encephalopathy
TBI: Traumatic brain injury
ICP: Intracranial pressure
SAH: Subarachnoid haemorrhage
GCS: Glasgow Coma Scale
BMI: Body mass index
MAP: Mean arterial pressure
ROC: Receiver operating characteristic
CHD: Coronary heart disease
ACI: Acute cerebral infarction
CH: Cerebral haemorrhage
MCA: Middle cerebral artery
SD: Standard deviation
AUC: Area under the curve
